# YOLO-FDCL: Improved YOLOv8 for Driver Fatigue Detection in Complex Lighting Conditions

**DOI:** 10.3390/s25154832

**Published:** 2025-08-06

**Authors:** Genchao Liu, Kun Wu, Wei Lan, Yunjie Wu

**Affiliations:** 1 School of Aeronautic Science and Engineering, Beihang University, Beijing 100191, China; 2 School of Aviation, Beihang University, Beijing 100191, China; 3 School of Automation Science and Electrical Engineering, Beihang University, Beijing 100191, China

**Keywords:** driver fatigue detection, YOLOv8, Mobilenetv4, RepFPN, multi-scale feature fusion

## Abstract

Accurately identifying driver fatigue in complex driving environments plays a crucial role in road traffic safety. To address the challenge of reduced fatigue detection accuracy in complex cabin environments caused by lighting variations, we propose YOLO-FDCL, a novel algorithm specifically designed for driver fatigue detection under complex lighting conditions. This algorithm introduces MobileNetV4 into the backbone network to enhance the model’s ability to extract fatigue-related features in complex driving environments while reducing the model’s parameter size. Additionally, by incorporating the concept of structural re-parameterization, RepFPN is introduced into the neck section of the algorithm to strengthen the network’s multi-scale feature fusion capabilities, further improving the model’s detection performance. Experimental results show that on the YAWDD dataset, compared to the baseline YOLOv8-S, precision increased from 97.4% to 98.8%, recall improved from 96.3% to 97.5%, mAP@0.5 increased from 98.0% to 98.8%, and mAP@0.5:0.95 increased from 92.4% to 94.2%. This algorithm has made significant progress in the task of fatigue detection under complex lighting conditions. At the same time, this model shows outstanding performance on our self-developed Complex Lighting Driving Fatigue Dataset (CLDFD), with precision and recall improving by 2.8% and 2.2%, respectively, and improvements of 3.1% and 3.6% in mAP@0.5 and mAP@0.5:0.95 compared to the baseline model, respectively.

## 1. Introduction

With the rapid advancement of socioeconomic development and accelerating urbanization, Advanced Driver Assistance Systems (ADASs) have emerged as a pivotal research focus in automotive engineering and artificial intelligence domains [1]. As a critical technological component of intelligent driving systems, driver fatigue state monitoring plays an essential role in traffic safety assurance. Statistical data from the U.S. transportation safety authorities indicate that fatigue-related driving incidents resulted in 633 fatalities in 2020 alone [2]. Comprehensive traffic accident investigations reveal that driver fatigue contributes to 8.8–9.5% of vehicle collisions, with this proportion escalating to 10.6–10.8% in crashes involving substantial property damage [3].

Driver fatigue refers to the physiological and psychological imbalance after long periods of driving, leading to decreased attention, slow reactions, and poor decision making. With the development of technology, driver fatigue is mainly detected through the following three methods.

Physiological signal-based methods require intrusive sensors but have demonstrated promising accuracy. Fu [4] explored ECG signals by utilizing cross-correlation between surface EMG and ECG peak factors for fatigue assessment. Oviyaa [5] proposed EEG sensor-embedded helmet systems that trigger automatic drowsiness alerts. Fan [6] employed prefrontal EEG analysis, extracting features such as energy and entropy for machine learning-based classification. Wu [7] created long-term memory EEG power maps that were subsequently fed into CNN-LSTM models for enhanced fatigue detection. While these methods achieve high accuracy, their intrusive nature and equipment requirements limit practical deployment.

Vehicle behavior-based methods offer non-intrusive monitoring but suffer from delayed response characteristics. Sandberg [8] utilized time series analysis to detect fatigue through driving indicators such as speed and steering wheel angle, employing neural networks trained via particle swarm optimization. Wang et al. [9] developed random forest models based on vehicle lateral and longitudinal accelerations combined with steering wheel angle measurements. The AutoVue system [10], developed by the I-teris company, detects lane deviation using highway-facing cameras for real-time monitoring. Ma Yongfeng et al. [11] investigated truck driver fatigue by analyzing travel speed data as key fatigue indicators. However, these approaches are constrained by individual driving habits and environmental conditions.

Computer vision-based methods have emerged as the most promising approach due to their non-intrusive nature, real-time capabilities, and cost-effectiveness. Zhang et al. [12] developed yawning detection systems integrating deep learning algorithms with Kalman filters and Track-Learning-Detection trackers. Hsu et al. [13] extracted the driver’s mouth and eye features for fatigue early warning using a deep CNN, achieving an average accuracy of 91.9%, though with slightly lower precision. Yan Zhu et al. [14] constructed a fatigue recognition framework utilizing least squares curve fitting for ocular and oral contours, incorporating statistical sequence-based CNN architectures. Xianguo L et al. [15] designed a YOLO-integrated algorithm for monitoring driver eye conditions, demonstrating 99.0% mAP performance on the CEW benchmark. Shugang Liu et al. [16] developed a dynamic tracking solution based on the YOLO architecture to identify small-scale fatigue indicators, effectively tackling facial feature degradation issues in practical driving scenarios.

Although existing driver fatigue detection methods achieve good performance under normal conditions, maintaining robust performance under complex lighting conditions remains a critical challenge. Real-world driving environments present varying lighting conditions—including strong light, weak light, sudden illumination changes, and glare interference—which severely degrade detection accuracy in current methods [17,18]. These dynamic lighting variations make it particularly challenging to reliably detect driver drowsiness states, significantly limiting the practical effectiveness of existing fatigue detection systems in ADAS applications. This paper presents YOLO-FDCL, an enhanced fatigue detection system specifically designed to address the complex lighting challenge. Our main contributions are as follows:

A MobileNetV4-enhanced backbone network that leverages the Universal Inverted Bottleneck (UIB) modules and MobileMQA attention mechanisms to improve feature extraction under varying illumination conditions, demonstrating superior performance in maintaining feature quality when traditional CNN backbones struggle with lighting-induced degradation in fatigue detection scenarios.

A novel RepFPN feature fusion architecture that uniquely integrates three complementary components—RepBlock reparameterization for efficient multi-branch learning, SimConv upsampling with enhanced nonlinear representation, and learnable Transpose modules for detail preservation—creating the first feature pyramid network specifically optimized for fatigue detection under complex lighting scenarios while achieving superior computational efficiency compared to existing FPN variants.

A comprehensive Complex Lighting Driving Fatigue Dataset (CLDFD) that addresses the critical gap in existing datasets by systematically simulating real-world lighting challenges through strategic data augmentation techniques. This dataset expands from 8849 to 44,245 images using targeted image sharpening, saturation adjustment, brightness variation, and color shifting, providing the first standardized benchmark for evaluating fatigue detection robustness across diverse lighting conditions encountered in practical driving scenarios, enabling comprehensive validation that was previously impossible with existing datasets.

The remainder of this paper is organized as follows: Section 2 reviews related work in fatigue detection; Section 3 presents our methodology; Section 4 describes experimental results; Section 5 concludes the paper.

## 2. Related Works

### 2.1. Computer Vision-Based Fatigue Detection

In early computer vision-based driver fatigue detection, most studies utilized single features for determining driver fatigue states. Alioua et al. [19] proposed a method using Support Vector Machine (SVM) and Circular Hough Transform (CHT) that achieved 98% accuracy in yawning recognition. Zhang and Wang [20] proposed an algorithm using image processing technology to process the images, which was evaluated using the SVM model. Finally, the sequence floating forward selection algorithm is used to select the optimal parameters, and the fatigue detection model was established. Zhuang et al. [21] proposed an eye opening and closing fatigue detection method based on pupil and iris segmentation. This method extracts eye images through the Dlib algorithm, uses a segmentation network to extract pupil and iris features from eye images, and employs a decision network to evaluate eye opening and closing degrees. Finally, PERCLOS values are calculated based on eye opening and closing degrees to predict driver states. This method was tested on the NTHUDDD dataset, achieving a fatigue detection accuracy of 96.72%. However, the aforementioned methods only perform fatigue detection through single facial fatigue features, resulting in poor accuracy and robustness. To address these issues, researchers proposed multi-feature fusion-based fatigue detection methods.

Zhao [22] proposed a fatigue detection method based on cascaded multi-task convolutional neural networks (MTCNNs) and eye-mouth convolutional neural networks (EM-CNNs). MTCNNs are is used for detecting and locating the driver’s facial area and facial landmarks. Then, EM-CNNs are used to classify the state of the eyes and the degree of mouth opening to assess the driver’s fatigue state. Guo [23] proposed a real-time efficient fatigue detection method based on YOLOV5, which achieved an average precision (mAP) of 75.44% on the BioID-Face dataset and 80.61% on the GI4E dataset. You et al. [24] constructed a driver fatigue recognition algorithm based on facial motion information entropy, employing an improved YOLOv3 tiny algorithm to locate driver facial information in various scenarios. The study used the Dlib algorithm combined with facial feature point coordinates to establish FFT (Face Feature Triangle) and generated facial feature vectors based on FFT information. Finally, facial motion information entropy was obtained through a sampler, and driver fatigue states were determined by comparing the information entropy with preset thresholds. Experimental results showed that this algorithm achieved a detection accuracy of 94.32%.

In summary, fatigue detection methods based on object detection can effectively identify driver fatigue states. However, existing models do not adequately consider the complex lighting environments in real-world cockpit scenarios, which leads to reduced accuracy and robustness. Therefore, to address these issues, this paper improves the YOLOv8s model and proposes a high-precision driver fatigue detection model YOLO-FDCL. First, this paper introduces the advanced MobileNetV4 to reconstruct the backbone network of the original model, optimizing the network structure and improving the feature extraction capability. Meanwhile, the neck network of YOLOv8s is modified by introducing the Rep-FPN feature fusion network, which effectively improves the inference speed and detection accuracy of the model while maintaining computational efficiency. Experimental results show that the model achieves high robustness and can accurately detect driver fatigue states in complex lighting environments.

### 2.2. The Challenge of Determining Actual Fatigue State

Despite significant advances in visual feature detection accuracy, determining a driver’s actual state of fatigue rather than merely detecting visual symptoms has emerged as the most critical challenge in recent years. As emphasized by Hu et al., “a single information cannot precisely reflect the actual state of the driver in different fatigue phases,” highlighting the inherent uncertainty in visual-based assessment methods [25]. This fundamental limitation stems from the complex, nonlinear relationship between observable facial features and actual driving impairment capacity.

The temporal dimension significantly compounds this challenge. EEG-based studies consistently demonstrate that cognitive fatigue manifests in brain activity patterns several minutes before becoming visually apparent [26]. This temporal lag creates a dangerous window where visual-based systems may provide false reassurance while drivers are experiencing diminished cognitive capacity. Furthermore, the progression from cognitive to visual fatigue is highly individual-dependent, with variations influenced by age, sleep debt, and individual fatigue resistance patterns [27].

Validation challenges arise from the absence of universally accepted ground truth for “dangerous” fatigue states. While subjective measures such as the Karolinska Sleepiness Scale provide standardized assessment, studies have documented significant discrepancies between self-reported sleepiness and objective performance metrics [28]. EEG-based methods, recognized as the “gold standard” for fatigue detection, demonstrate superior accuracy but require intrusive sensors impractical for widespread deployment [29]. Multi-modal approaches combining visual features with physiological signals have shown improved reliability but face complexity and cost barriers for commercial adoption [30].

The threshold-setting problem represents perhaps the most critical unsolved challenge in practical deployment. Unlike typical computer vision applications, fatigue detection systems must balance dramatically different risk profiles: false negatives can result in accidents, while false positives may lead to system abandonment [31]. Environmental factors including varying lighting conditions and the transition from laboratory to real-world settings introduce additional uncertainties that current literature has inadequately addressed. As noted in recent reviews, “the gap between achieving high detection accuracy in controlled settings and providing reliable fatigue warnings in diverse real-world conditions represents the next frontier in driver safety research” [32]. These considerations highlight the need for approaches that improve visual detection robustness while acknowledging the inherent uncertainties in translating visual observations to functional safety assessments.

### 2.3. Feature Fusion

Deep learning-based object detection methods typically rely on backbone networks to extract features, but effective representation requires capturing both fine-grained details and semantic information across multiple scales. Lin et al. [33] developed the Feature Pyramid Network (FPN), which strengthens feature representation by integrating low-level high-resolution features with high-level semantic features through top-down pathways and lateral connections.

Building upon FPN, advanced architectures including PANet [34], NAS-FPN [35], ASFF [36], BiFPN [37], and AugFPN [38] have been proposed to further enhance detection performance through improved multi-scale feature fusion strategies.

These studies demonstrate that multi-scale feature fusion is crucial for robust object detection, particularly in challenging lighting environments. Different feature levels exhibit varying sensitivities to illumination changes: shallow layers capture details that may be affected by shadows and glare, while deeper layers extract semantic features more robust to lighting variations. Multi-scale fusion enables networks to compensate for lighting-induced degradation by leveraging complementary information across scales, maintaining detection accuracy under adverse lighting conditions. However, existing FPN variants often suffer from computational overhead and suboptimal feature integration under extreme lighting variations. Therefore, we propose Rep-FPN, which employs reparametrized convolutions to enhance feature fusion efficiency while providing stronger multi-scale feature integration specifically optimized for complex lighting scenarios, enabling more robust fatigue detection with improved computational efficiency.

## 3. Methods

### 3.1. YOLOv8 Model

**Figure 1:**
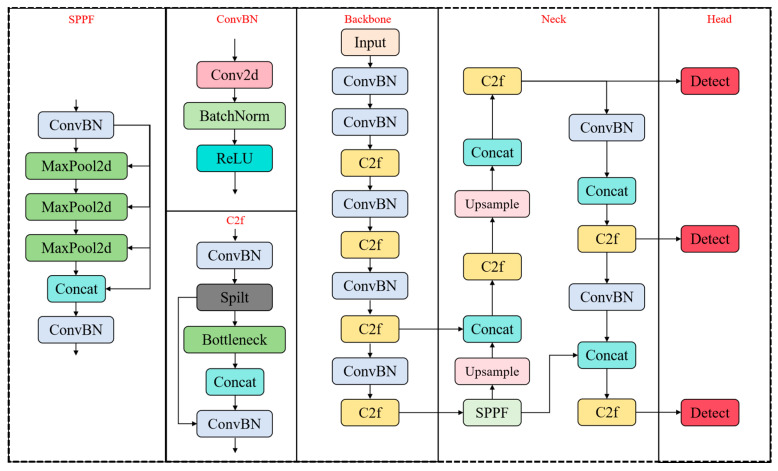
The network structure of YOLOV8.

The YOLOv8 model architecture consists of four components: Backbone, Neck, and Detection Head. Figure 1 illustrates the structure of the YOLOv8 network.

- Input Layer:

The input layer adjusts the dimensions of the input images to meet training requirements. It also performs operations such as scaling, tone adjustments, and Mosaic data augmentation to enhance the diversity of the training data.

- Backbone Network:

The backbone network adopts a Cross-Stage Partial Network (CSPNet) architecture, which is designed to enhance feature extraction capabilities. It improves overall model performance by balancing computational efficiency and feature representation capacity.

- Neck:

The neck performs feature fusion, employing both the Feature Pyramid Network (FPN) and Path Aggregation Network (PAN). By integrating features across different scales, these networks enhance the model’s ability to detect objects in diverse sizes and positions.

- Detection Head:

The detection head adopts a decoupled structure, dividing the detection and classification tasks. This separation reduces the model’s parameter count and complexity, which in turn boosts its generalization and robustness. It outputs confidence scores and position details for objects, facilitating accurate detection.

### 3.2. YOLO-FDCL Detection Model

This paper proposes a YOLO-FDCL model based on the YOLOv8s series, which is specialized for driver fatigue detection. The model improves the detection accuracy and, at the same time, enhances the detection capability of the model for complex lighting environments.

**Figure 2:**
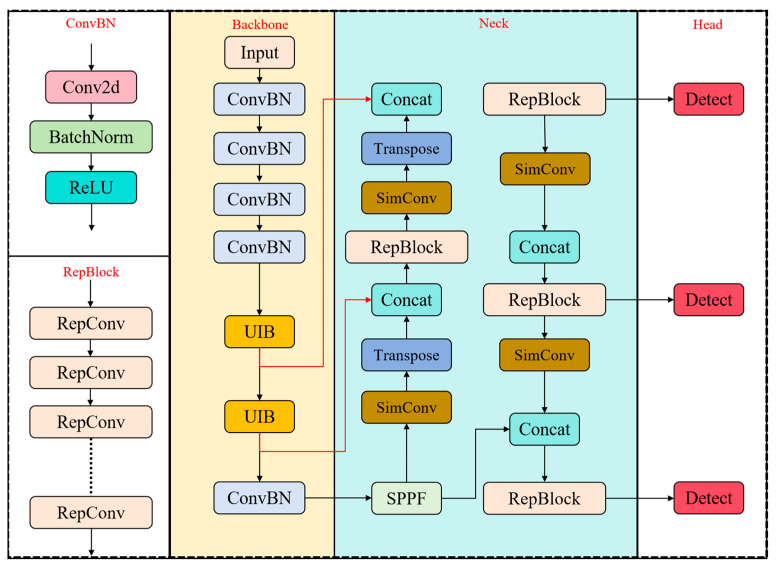
The network structure of YOLO-FDCL.

The overall structure of YOLO-FDCL is shown in Figure 2. The overall with YOLO-FDCL is similar to the overall structure of YOLOv8, which is mainly composed of backbone, neck, and head. In order to solve the complex driving noise (different driving habits of drivers, complex lighting environment while driving) on the network to extract features interference, this study selected the MobileNetv4 network [39] as the backbone network, which effectively improves the feature extraction ability of the model, as well as the robustness of the network.

At the same time, this paper refers to the RepVGG algorithm to modify the network’s neck layer to add a reparameterization block (RepBlock) [40] to form the unique feature pyramid structure of this paper can effectively improve the feature fusion ability to improve the accuracy and speed of the driver fatigue detection.

RepBlock is a network module designed based on the re-parameterization concept, consisting of multiple stacked or combined RepConv units with enhanced feature representation capabilities and optimized inference performance. Each RepConv unit utilizes a multi-branch structure during the training phase to achieve diverse feature learning. These branches include a 3 × 3 convolution branch as the main branch, along with auxiliary 1 × 1 convolution, Batch Normalization (BN), and identity mapping branches. The multi-branch structure independently learns parameters during training, effectively enhancing the model’s feature representation ability and generalization capability, thus better adapting to complex task requirements. During the inference phase, all branch parameters are mathematically re-parameterized and fused into an equivalent 3 × 3 convolution operation.

This design significantly reduces the computational overhead during inference while preserving the feature expression effects learned during training. The overall structure of RepBlock, through the stacking of multiple RepConv units, further improves the network’s feature extraction capabilities. Its re-parameterization process ensures higher efficiency and optimal accuracy during inference.

#### 3.2.1. Improved MobileNetv4 Backbone

The traditional YOLOv 8 backbone uses the CSPDarknet architecture to extract multi-scale and high-level features through multiple convolutions and pooling, but it is a deep architecture, and complex convolutions consume a lot of computing resources, limiting deployment on low-performance hardware. In addition, the feature extraction ability of this architecture is slightly insufficient in response to fatigue detection under complex lighting conditions, so we introduce the Mobilenetv4 network as the backbone network of YOLO-FDCL, which can effectively improve the feature extraction ability of the model while realizing the lightweight of the network.

**Figure 3:**
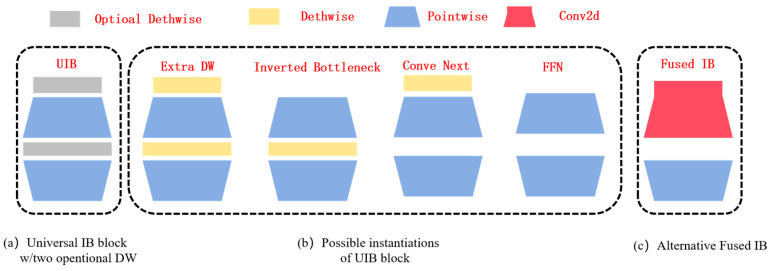
The network structure of UIB.

MobileNetV4 is an efficient neural network architecture designed for mobile devices. As shown in Figure 2, it demonstrates excellent performance on various hardware platforms by integrating the Universal Inverted Bottleneck (UIB) and MobileMQA attention mechanisms. The feed-forward network (FFN) by the UIB module unifies the Inverted Bottleneck (IB), ConvNext, and Visual Transformer (VIT), and introduces the Extra Depth IB (ExtraDW) block, which integrates the strengths of the above modules to increase the network’s depth and sense field and maximize the computational utilization at less computational cost. The possible architecture of the universal UIB is shown in Figure 3 below.

Compared with the traditional inverted bottleneck structure, the UIB module adopts two deep convolutions before the expansion layer and between the expansion layer and the projection layer, so that the clever structural design can achieve more detailed adjustment of the feature extraction process, so as to improve the flexibility of feature extraction and meet the needs of different tasks. Based on these two variable depth convolutions, the UIB module can form four different instances, namely Extra dw, Inverted bottleneck, Conv next, and FFN (Feed Forward Network). Through two deep convolutions, Extra dw can capture deeper spatial features and extract these features at different levels, effectively enhancing the depth and receptive field of the network to meet the input and output requirements of the network. Inverted bottleneck increases the capacity of the model by performing a spatial blending operation after expanding the feature. Conv next performs the blending of spatial information in advance before the feature map expansion, making the spatial blending of larger convolution kernels more efficient. The FFN module consists of two point-by-point convolutions, and inserts an activation function and a normalization layer between the two layers, which is suitable for the global feature integration of the network backend. Through the information fusion between spatial features and channel features, the UIB module can adaptively adjust the size of the sensing field of the model and efficiently use computing resources, thereby improving the performance of the model. The four possible UIB module instances and built UIB blocks are shown in Figure 3 below.

The module also introduces mobile MQA, which is an optimizer for the attention module and improves inference speed by more than 39%. Unlike traditional Multi-Headed Self-Attention (MHSA), Mobile’s MQA shares keys and values across all heads, reduces memory access requirements while maintaining high-resolution queries through asymmetric spatial subsampling, and significantly improves computational efficiency. The overall network architecture of the MobileNetV4 feature extraction backbone used in this paper is shown in Table 1. YOLO-FDCL efficiently extracts driver fatigue features in complex lighting environments using UIB, while mobile MQA attention further reduces the interference of background noise, thus achieving efficient extraction of fatigue features.

#### 3.2.2. Design of the Neck Section-RepFPN

**Figure 4:**
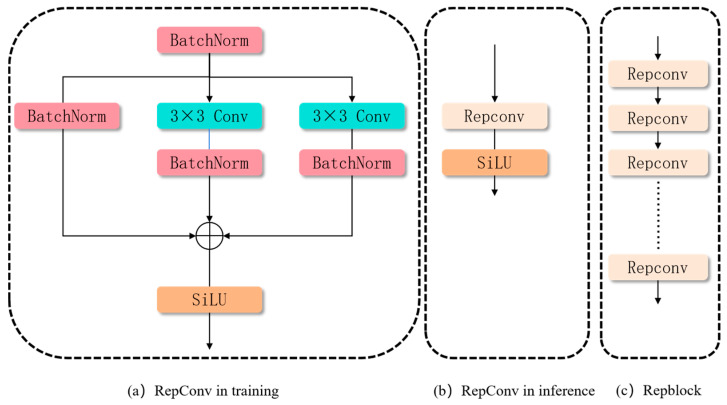
Repconv training as well as inference schematics, and RepBlock structure diagrams.

Although the traditional YOLOv8 feature gold tower structure can effectively integrate multi-scale features, in strong backlight, direct sunlight or low light environments, the traditional FPN may not be able to effectively capture key facial features such as eyes and mouth due to overexposure or underexposure, resulting in false detection and increased missed detection rate of the model. At the same time, under low-light conditions, it is difficult for the traditional FPN downsampling layer to capture enough detailed features, which limits the overall detection accuracy. To this end, we proposed RepFPN to improve the robustness of the model. RepFPN is mainly composed of three parts: Repblock, simconv, and Transpose. Here, we refer to the idea of structural reparameterization proposed by Ding et al. (during training, the network retains multiple branches to learn more subsets. In the inference process, the network merges multiple branches into one path, thereby improving the inference speed of the model), and proposes a reparameterization module (Repblock) in the structure of the model, as shown in Figure 4 below, to improve the inference speed of the model and the information extraction ability of features.

The reparameterization procedure is explained below. As shown in Equation (1), the output *Q_out_* of RepConv is calculated without activation as follows:

(1) $$\begin{array}{l} Q_{out}=BN(Q_{in},\mu_{id},\omega_{id},\varepsilon_{id},\delta_{id})\\ \;\;\;+BN(Q_{in}*\mu_{K1},\omega_{K1},\varepsilon_{K1},\delta_{K1})\\ \;\;\;+BN(Q_{in}*\mu_{k3},\omega_{k3},\varepsilon_{k3},\delta_{k3}) \end{array}$$

In this context, *D_k_*_1_ and *D_k_*_3_ denote the weights of the 1 × 1 and 3 × 3 convolutions, respectively. We use *μ_k_*_3_, *ω_k_*_3_, *ε_k_*_3_, *δ_k_*_3_ to denote the accumulated mean, standard deviation, learned scaling factor, and bias of the Batch Normalization (BN) after the 3 × 3 convolution. Similarly, *μ_k_*_1_, *ω_k_*_1_, *ε_k_*_1_, *δ_k_*_1_ are utilized for the 1 × 1 convolution, while *μ_kd_*, *ω_kd_*, *ε_kd_*, *δ_kd_* are designated for the identity branch.

The input feature map is denoted by *Q_in_*. The identity branch can be treated as a 1 × 1 branch with a constant weight of 1, and the 1 × 1 convolution can be equivalently considered a 3 × 3 convolution by zero-padding. Next, we show the reparameterization process using the 3 × 3 branch as an example:

(2) $$\begin{array}{l} BN(Q_{in}*D_{k3},\mu_{k3},\omega_{k3},\varepsilon_{k3},\delta_{k3})\\ =(Q_{in}*D_{k3}-\mu_{k3})\frac{\varepsilon_{K3}}{\omega_{K3}}+\delta_{K3}\\ =Q_{in}*(\frac{Q_{K3}\gamma_{K3}}{\omega_{K3}})-\frac{\mu_{K3}\varepsilon_{K3}}{\omega_{K3}}+\delta_{K3} \end{array}$$

As a result, the weight and bias for the 3 × 3 branch can be transformed into $D_{K3}^{\prime }=D_{K3}\varepsilon_{K3}/\omega_{K3}$ and $a_{K3}^{\prime }=\mu_{K3}\varepsilon_{K3}/\omega_{K3}+\delta_{k3}$. A similar derivation applies to the other two branches. The final output of RepConv during inference is represented by Equation (3).

(3) $$\begin{array}{l} D=SiLU(D_{out})=SiLU(D_{in}*D^{\prime }+b^{\prime })\\ D^{\prime }=D_{k3}^{\prime }+D_{k1}^{\prime }+D_{id}^{\prime }\\ a^{\prime }=a_{k3}^{\prime }+a_{k1}^{\prime }+a_{id}^{\prime } \end{array}$$

where *Q* is the output of RepConv with SiLU activation function; $D_{k1}^{\prime }$ and $D_{id}^{\prime }$ are the weights of 1 × 1 convolution and identity branch, respectively; $a_{k1}^{\prime }$ and $a_{id}^{\prime }$ are the bias of 1 × 1 convolution and identity branch, respectively. In this case, Q denotes the output of RepConv with the SiLU activation function, and $D_{k1}^{\prime }$, $D_{id}^{\prime }$ are the weights for the 1 × 1 convolution and identity branch, respectively. $a_{k1}^{\prime }$ and $a_{id}^{\prime }$ are the biases for these respective branches.

Although the RepConv structure design helps reduce the model’s parameter count and improve inference speed, a single RepConv module is insufficient to effectively capture subtle facial expression changes in drivers under complex lighting conditions, especially when fatigued. Therefore, by cascading multiple RepConv modules to form a RepBlock, the model’s depth is increased, enhancing its generalization ability and flexibility. This enables the model to extract deeper semantic information from feature maps, thus improving detection accuracy and inference speed while reducing the model’s parameter count.

Secondly, we will replace the upsampling operation in the original model with SImconv and the Transpose module. Simconv mainly contains three operations, Conv2d, batch normalization operation, and Relu activation function, and the custom Simconv replaces the original Silu layer with the Relu layer, which enhances the nonlinear representation ability of the model. By adjusting the number of channels and feature representations of the feature map, Simconv can more effectively handle the complex features caused by lighting changes, especially in low-light or high-light environments, and still accurately extract the driver’s subtle features (such as eye closures, etc.). Transpose is a learnable upsampling method, which can learn more complex feature mapping relationships than the original closest collar or interpolation upsampling methods, especially under complex lighting conditions. This learnable upsampling method avoids the image distortion or blurring problems that may be brought about by traditional methods, so that the details of the feature map can be better preserved and enhanced, and the accuracy of driver fatigue detection is further improved. By optimizing the inference speed, enhancing the nonlinear expression ability and improving the feature upsampling method, RepFPN effectively improves the driver fatigue detection ability in complex lighting environments.

### 3.3. Fatigue Detection Mechanism

Unlike traditional fatigue detection methods that rely on explicitly extracted facial features such as PERCLOS (Percentage of Eye Closure), eye aspect ratio (EAR), or mouth aspect ratio (MAR), our YOLO-FDCL model adopts an end-to-end deep learning approach that directly learns fatigue patterns from holistic facial images. Our model processes the entire facial region as a unified input without requiring manual feature engineering or specific facial landmark detection. The deep convolutional architecture automatically learns discriminative features that distinguish between fatigue and non-fatigue states through several key mechanisms.

The MobileNetV4 backbone extracts hierarchical features from the entire facial image, capturing both local details and global patterns that correlate with fatigue states. Rather than explicitly detecting eye closure or yawning, the model learns implicit representations of fatigue through supervised training on labeled data. These learned features may include subtle changes in facial muscle tension, head pose variations, and overall facial expressions that humans might not easily quantify. The model classification is based on the annotation standards defined in our datasets, where facial images in the YAWDD dataset are categorized into “drowsy” and “undrowsy” states based on observable driver behaviors. The labeling criteria follow established fatigue indicators but are applied at the image level rather than feature level, with no explicit facial region segmentation or feature point detection performed.

This holistic approach offers several advantages. By learning from the entire facial region, the model is less susceptible to partial occlusions or failures in specific feature detection, providing enhanced robustness. The model can capture complex fatigue patterns that might not be captured by predefined features, demonstrating superior adaptability. Additionally, this approach eliminates the computational overhead of multiple feature extractors and fusion mechanisms, resulting in improved efficiency. While traditional approaches decompose the fatigue detection task into multiple sub-problems (eye detection → eye state classification → PERCLOS calculation), our YOLO-FDCL model directly maps facial images to fatigue states, allowing the deep neural network to discover the most discriminative features automatically. This end-to-end learning paradigm is particularly advantageous under complex lighting conditions, where traditional feature extraction methods often fail due to poor visibility of specific facial landmarks.

## 4. Experiments and Results

This section presents comprehensive experimental validation of our YOLO-FDCL model through multiple complementary analyses. Our evaluation framework systematically demonstrates the effectiveness, robustness, and practical applicability of our approach across diverse scenarios and challenging lighting conditions.

The experimental validation proceeds through three main phases. First, we describe our datasets, including the novel Complex Lighting Driving Fatigue Dataset (CLDFD), experimental setup, and evaluation metrics (Section 4.1, Section 4.2, Section 4.3 and Section 4.4). Second, we conduct comparative analysis against state-of-the-art detection methods and systematic ablation studies to validate individual component contributions (Section 4.5 and Section 4.6). Finally, we perform specialized validation under complex lighting environments and provide visual analysis demonstrating model attention mechanisms (Section 4.7 and Section 4.8).

This multi-faceted approach ensures thorough validation of our claims regarding improved fatigue detection accuracy and robustness under challenging lighting conditions encountered in real-world driving scenarios.

### 4.1. Datasets

**Figure 5:**
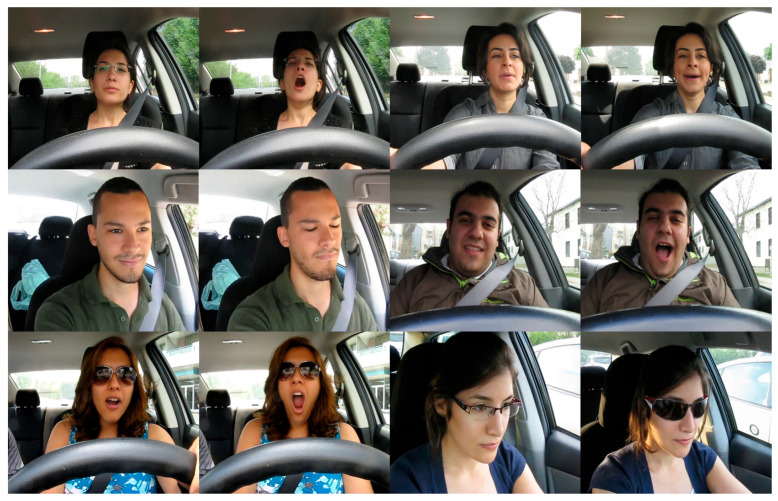
Sample frames from the YAWDD dataset.

To comprehensively evaluate our YOLO-FDCL model’s performance on driver fatigue detection, we utilize multiple datasets that provide diverse and challenging scenarios. The YAWDD (yawning detection dataset) [41] is the main dataset for us to verify the performance of fatigue detection, and some images of the dataset are shown in Figure 5 below. This dataset comprises videos of drivers’ faces captured under various lighting conditions and is organized into two distinct sets based on camera placement. The first set contains 322 videos recorded with cameras mounted under the front and rearview mirrors, featuring 3–4 videos per participant, while the second set consists of 29 videos captured from dashboard-mounted cameras. The dataset encompasses diverse participant demographics, including both male and female drivers of different ethnicities, with varying eyewear conditions (no glasses, regular eyeglasses, and sunglasses). All videos are in AVI format at 640 × 480 resolution with 30 fps, and each video captures three distinct states: (1) normal driving without talking, (2) talking or singing, and (3) yawning. From this dataset, we extracted a total of 10,611 images, which were divided into training and validation sets at an 8:2 ratio.

The UTA-RLDD (University of Texas at Arlington Real-Life Drowsiness Dataset) [42] provides complementary data with its unique naturalistic approach. This dataset contains approximately 30 h of RGB videos from 60 healthy participants (51 men and 9 women) representing diverse ethnic backgrounds: 10 Caucasian, 5 non-white Hispanic, 30 Indo-Aryan and Dravidian, 8 Middle Eastern, and 7 East Asian individuals, aged 20–59 years. Unlike controlled laboratory recordings, participants self-recorded videos using their personal cell phones or web cameras in their chosen indoor environments, resulting in authentic variations in backgrounds, camera angles, and recording quality. Each participant provided three 10 min videos corresponding to different alertness levels (alert, low vigilant, and drowsy) based on the Karolinska Sleepiness Scale, totaling 180 videos with frame rates below 30 fps.

To specifically address the challenge of fatigue detection under complex lighting conditions and validate our model’s robustness, we created the Complex Lighting Driving Fatigue Dataset (CLDFD). This custom dataset was constructed through careful integration and augmentation of samples from both YAWDD and UTA-RLDD datasets. Our dataset construction process involved three key steps. First, we strategically selected 8849 facial images from both source datasets, prioritizing samples that represent diverse lighting conditions, participant characteristics, and fatigue states. Second, we applied comprehensive image enhancement techniques, including image sharpening, saturation adjustment, brightness variation, and color shifting, to simulate the challenging lighting conditions encountered in real-world driving scenarios such as strong backlighting, direct sunlight, shadows, and low-light environments. Through these augmentation techniques, we expanded the dataset to 44,245 images, ensuring adequate representation of various lighting challenges.

**Figure 6:**
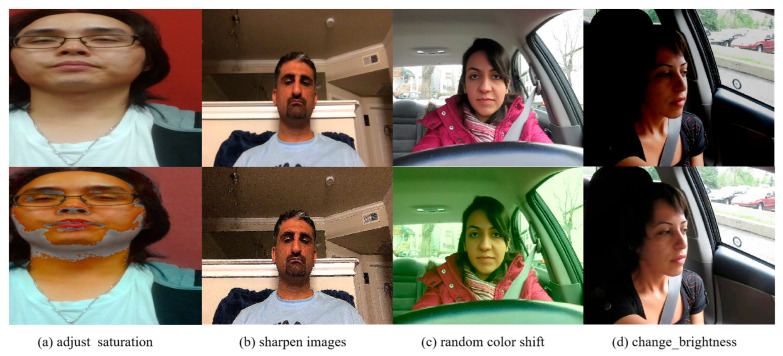
Sample frames from the CLDFD.

For annotation consistency, we employed the LabelImg tool to manually label each image according to two primary fatigue states: “drowsy” and “undrowsy” (alert). Our annotation protocol followed strict classification standards to ensure accurate correspondence between visual fatigue indicators and assigned labels. Multiple annotators reviewed ambiguous cases to maintain labeling quality. The final dataset was divided into training, validation, and test sets at a ratio of 8:1:1, providing sufficient data for both model training and comprehensive evaluation. Sample images from CLDFD, shown in Figure 6, demonstrate the variety of lighting conditions and fatigue states represented in our dataset.

### 4.2. Experimental Environment

This study was conducted in an environment featuring a 13th Gen Intel Core i9-13900H processor, 32 GB of RAM, and an NVIDIA GeForce RTX 4070 GPU with 8 GB of VRAM. The software environment consisted of Python 3.8, Windows 11 operating system, and PyTorch 2.2.2 deep learning framework. The detailed configurations are listed in Table 2.

### 4.3. Training Parameters

The training strategy we employ follows a similar pattern to YOLOv8, using the stochastic gradient descent (SGD) optimizer with momentum and applying cosine annealing for learning rate adjustments. The initial learning rate is set to 0.01. We apply traditional data augmentation methods, including Mosaic, Mixup, and random flipping. The model was trained for 300 epochs on an RTX 4070, with a batch size of 16, and trained from scratch on the YAWDD dataset. For training, we set the size of each input image 640 × 640.

### 4.4. Evaluating Indicator

To evaluate the detection performance of the proposed improved model YOLO-FDCL, this paper uses the mean average precision (mAP_@0.5_) at an IoU threshold of 0.5, and the mean average precision (mAP_@0.5:0.95_) over the IoU range of 0.5 to 0.95 as metrics for model detection accuracy. The number of parameters and GFLOPs are used as indicators of model complexity. These metrics effectively reflect the performance of the model in terms of both accuracy and complexity in the driver fatigue detection task.

Precision (P) refers to the fraction of true positive samples among those predicted as positive by the model, and is mathematically defined as follows:

(4) $$\mathrm{Precision}=\frac{TP}{TP+FP}$$

where True Positives (TPs) represent the number of correctly predicted positive samples, and False Positives (FPs) represent the number of incorrectly predicted positive samples.

The Mean Average Precision (mAP) is the average of the precision values across different classes and various IoU (Intersection over Union) thresholds. First, for each class and predicted box, whether it is a correct detection is determined based on its IoU with the ground truth box. Then, the area under the Precision–Recall curve was calculated for each class, and finally, these areas were averaged across all classes. The mathematical expression is as follows:

(5) $$mAP=\frac{1}{N_{c}}\sum_{i=1}^{N_{C}}AP_{i}$$

where *Nc* is the total number of classes, and *AP_i_* is the average precision for the *i*-*th* class.

### 4.5. Comparisons

To evaluate the performance of the YOLO-FDCL model, we compared it with multiple mainstream models, including the YOLOv8 series, YOLOv10, Faster RCNN, Dynamic RCNN [43], RT-DETR [44], and Deformable-DETR [45]. Training was performed on the YAWDD dataset while maintaining consistency of individual training parameters and hyperparameters, and the training results are shown in Table 3.

From the results in Table 3, it can be seen that YOLO-FDCL demonstrates excellent performance on key metrics, including precision, recall, mAP@0.5, and mAP@0.5:0.95. Specifically, YOLO-FDCL achieves a precision of 98.8%, a recall of 97.5%, a mAP@0.5 of 98.8%, and a mAP@0.5:0.95 of 94.2%. These values are all superior to the comparison models. For example, compared to the classic two-stage detection models Faster R-CNN and Dynamic R-CNN, YOLO-FDCL not only outperforms them in terms of accuracy and recall, but also features a more compact model with significantly reduced computational overhead and notably lower FLOPs.

Compared to traditional lightweight models such as YOLOv5-S and YOLOv10-S, YOLO-FDCL not only outperforms them in precision and recall, but also improves mAP@0.5 by 0.6% for both models, and achieves improvements of 2.7% and 2.8%, respectively, on the more stringent mAP@0.5:0.95 metric, while maintaining similar computational overhead.

Within the YOLOv8 series, YOLO-FDCL demonstrates comprehensive performance advantages. Compared to the baseline model YOLOv8-S, YOLO-FDCL achieves improvements of 1.4%, 1.2%, 0.8%, and 1.9% in precision, recall, mAP@0.5, and mAP@0.5:0.95, respectively, while slightly reducing the parameter count.

Compared to other YOLOv8 variants, YOLO-FDCL also performs excellently. Compared to the lightweight YOLOv8-N, although the parameter count has increased, all performance metrics show significant improvements. Compared to the medium-scale YOLOv8-M and large-scale YOLOv8-L, YOLO-FDCL not only surpasses them in detection accuracy, but also significantly reduces computational complexity—compared to YOLOv8-M, the parameter count is reduced by half and FLOPs are reduced by a factor of 2, and compared to YOLOv8-L, resource consumption is even more dramatically reduced.

It is worth noting that although the largest YOLOv8-X model has slightly higher recall than YOLO-FDCL (97.9% vs. 97.5%), this minor difference is a reasonable design trade-off. Our model design philosophy is to maintain high detection accuracy while significantly reducing computational complexity and model size to meet the practical requirements of resource-constrained scenarios such as in-vehicle systems. YOLO-FDCL has only 10.96 M parameters and 32.3G FLOPs, compared to YOLOv8-X’s 61.60 M parameters and 226.7G FLOPs, demonstrating significant advantages in computational efficiency. This indicates that YOLO-FDCL successfully achieves the dual goals of performance improvement and efficiency optimization.

Furthermore, compared to the Transformer-based model RT-DETR (precision 98.3%, recall 95.6%, mAP@0.5 96.5%, FLOPs 103.4G), YOLO-FDCL not only achieves 2.3% higher mAP@0.5 and 4.4% higher mAP@0.5:0.95, but also reduces computational complexity by 3.2 times. This balance between maintaining high accuracy and achieving high efficiency makes YOLO-FDCL particularly suitable for deployment on in-vehicle embedded devices.

**Figure 7:**
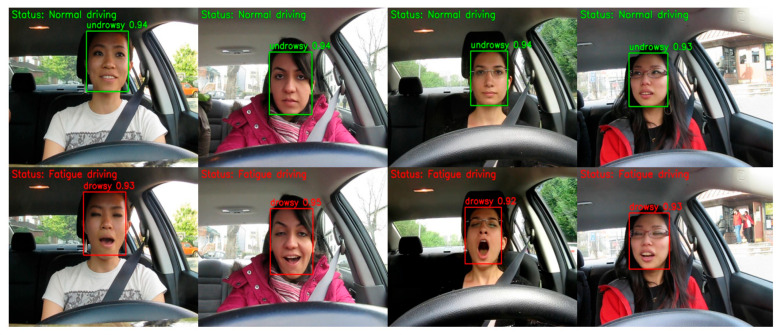
YOLO-FDCL detection results.

In automotive safety applications such as driver fatigue detection, ensuring driver safety is the primary goal. Experimental results demonstrate that YOLO-FDCL performs excellently in both accuracy and recall, capable of precisely identifying driver fatigue states while effectively reducing false positives and false negatives. This reliable detection performance enables timely warnings at critical moments, providing a more practical and trustworthy technical solution for automotive safety systems. Figure 7 shows partial results of driver fatigue detection on the YAWDD dataset, further confirming the accuracy of this model’s detection.

### 4.6. Ablation Experiments

#### 4.6.1. Backbone Ablation Experiment

In order to verify the performance improvement effect of MobileNetV4-backbone in the driver fatigue detection model based on YOLO-FDCL, the original YOLOv8 backbone model was replaced with other lightweight (such as Fasternet, EfficientVIT, Starnet, timm backbone), and the YOLO-FDCL model using MobileNetV4-backbone was compared under the same test conditions. The experimental results are shown in Table 3. As can be seen from the experimental results in Table 4, the YOLO-FDCL model using MobileNetV4 as the backbone network shows significant advantages in several key strict metrics. Compared to the relatively new lightweight backbones Fasternet, EfficientVIT, and Starnet, the map50 increases by 0.6%, 0.5%, and 0.4%, respectively. The more stringent experimental requirements of map50:95 increase by 1%, 1%, and 0.8%, respectively. Meanwhile, compared to the Transformer series backbone TIMM, this model improves by 0.6% and 1.1% on map50 and map95, respectively.

#### 4.6.2. FPN Ablation Experiment

Similarly, to validate the performance improvement of RepFPN in the YOLO-FDCL model, the original YOLOv8 NECK part was replaced with other common feature pyramid structures (such as BiFPN, MAFPN, HSFPN), and the YOLO-FDCL model using RepFPN was compared under the same testing conditions. The specific experimental results are shown in Table 5. From the experimental results, we can see that models using the RepFPN structure have higher accuracy compared to those using other traditional FPN structures, with improvements of 1%, 1.2%, and 0.9% on map50:95, respectively.

#### 4.6.3. Integral Ablation Experiment

To validate the effectiveness of the proposed YOLO-FDCL model for driver fatigue detection under complex lighting conditions, an ablation study was conducted. The original YOLOv8s network and its improved versions with each enhanced module were tested, and the experimental results are shown in Table 6.

When the backbone network is substituted with the Mobilenetv4 model, the precision, recall, mAP@0.5, and mAP@50:95 improve by 0.9%, 0.5%, 0.8% and 0.9%, respectively. These performance improvements stem from Mobilenetv4’s advanced architectural design. Mobilenetv4 employs optimized depthwise separable convolutions and Universal Inverted Bottleneck (UIB) modules to more effectively extract multi-scale features while reducing information loss. Additionally, its efficient attention mechanism adaptively focuses on critical facial regions, including eyes and mouth, ensuring precise localization of key fatigue detection features even under uneven illumination conditions.

The integration of RepFPN in the neck architecture yields precision, recall, mAP@0.5, and mAP@50:95 improvements of 1.2%, 0.1%, 0.7% and 1.0%, respectively, while achieving an 11% reduction in parameter count. These improvements stem from the synergistic effect of RepFPN’s three core components. RepBlock learns rich feature representations through a multi-branch structure during training and reparameterizes into equivalent 3 × 3 convolutions during inference to enhance efficiency. The SimConv module replaces traditional upsampling with its Conv2d-BN-ReLU structure enhancing nonlinear expression capabilities to better handle complex features caused by lighting variations. Transpose learnable upsampling avoids image distortion issues of traditional interpolation methods, better preserving and enhancing feature map details under complex lighting conditions, thereby significantly improving precision performance.

The synergistic integration of Mobilenetv4 backbone and RepFPN neck achieves optimal performance improvements of 1.4%, 1.2%, 0.8%, and 1.8% in precision, recall, mAP@0.5 and mAP@50:95, respectively. This synergistic effect stems from the complementarity of the two components in the feature processing pipeline: Mobilenetv4 is responsible for extracting rich multi-scale feature representations from input images, with its UIB modules and MQA mechanism ensuring discriminative ability even under adverse lighting conditions; while RepFPN optimally integrates these multi-scale features through its reparameterized feature fusion strategy. This end-to-end optimization enables the entire network to maintain high-precision detection while enhancing sensitivity to subtle fatigue signs when facing complex lighting challenges, achieving balanced improvements in both precision and recall.

### 4.7. Lighting Environment Experiment

To comprehensively evaluate the robustness and generalization capability of YOLO-FDCL under challenging lighting conditions encountered in real-world driving scenarios, we conducted specialized experiments on our self-constructed Complex Lighting Driving Fatigue Dataset (CLDFD). While the YAWDD dataset provides a valuable benchmark for fatigue detection research, its limited representation of diverse lighting conditions necessitates additional validation under more challenging and realistic lighting scenarios. The CLDFD, as described in Section 4.1, was specifically designed to address this limitation by incorporating systematic data augmentation techniques that simulate various lighting challenges, including strong backlighting, direct sunlight exposure, shadow variations, and low-light conditions. This dataset expansion from 8849 to 44,245 images ensures comprehensive coverage of lighting conditions that drivers encounter in real-world scenarios. The comparative experimental results on the CLDFD are presented in Table 7, where we evaluate both the baseline YOLOv8-S and our proposed YOLO-FDCL under these challenging conditions.

The experimental results demonstrate that YOLO-FDCL maintains superior performance even under the more challenging lighting conditions represented in the CLDFD. Specifically, our model achieves significant improvements of 2.8% in precision (89.7% vs. 86.9%), 2.2% in recall (88.6% vs. 86.4%), 3.1% in mAP@0.5 (90.5% vs. 87.4%), and 3.6% in mAP@0.5:0.95 (84.6% vs. 81.0%) compared to the baseline YOLOv8-S model.

These improvements are particularly critical for safety-critical fatigue detection applications. The enhanced precision reduces false alarms that could lead to driver distraction or system disabling, while the improved recall ensures fewer missed fatigue events—a crucial factor since undetected drowsiness poses significant safety risks. In complex lighting scenarios where traditional systems often fail, maintaining high precision and recall becomes even more vital as drivers are already challenged by poor visibility conditions that increase accident risk. The consistent performance gains across all metrics under challenging lighting conditions validate that YOLO-FDCL possesses the robustness necessary for reliable fatigue detection in real-world driving scenarios.

### 4.8. Visual Analysis

**Figure 8:**
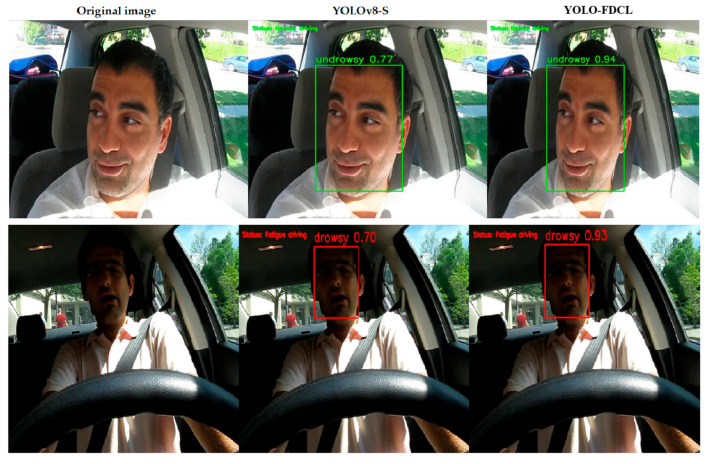
Comparing the performance results of the YOLOv8-S and YOLO-FDCL algorithms.

Figure 8 presents a comparison of the YOLOv8s algorithm and the proposed YOLO-FDCL algorithm for the fatigue detection task under complex lighting conditions. The complex lighting conditions and the exposure of the driver’s face to variable lighting environments during driving make it more challenging for the model to extract effective facial fatigue features. In typical detection scenarios, YOLO-FDCL surpasses the original YOLOv8s network in accuracy and exhibits enhanced robustness. In the figure, the green and red boxes correspond to anchor boxes, and the numbers within them denote the confidence scores.

**Figure 9:**
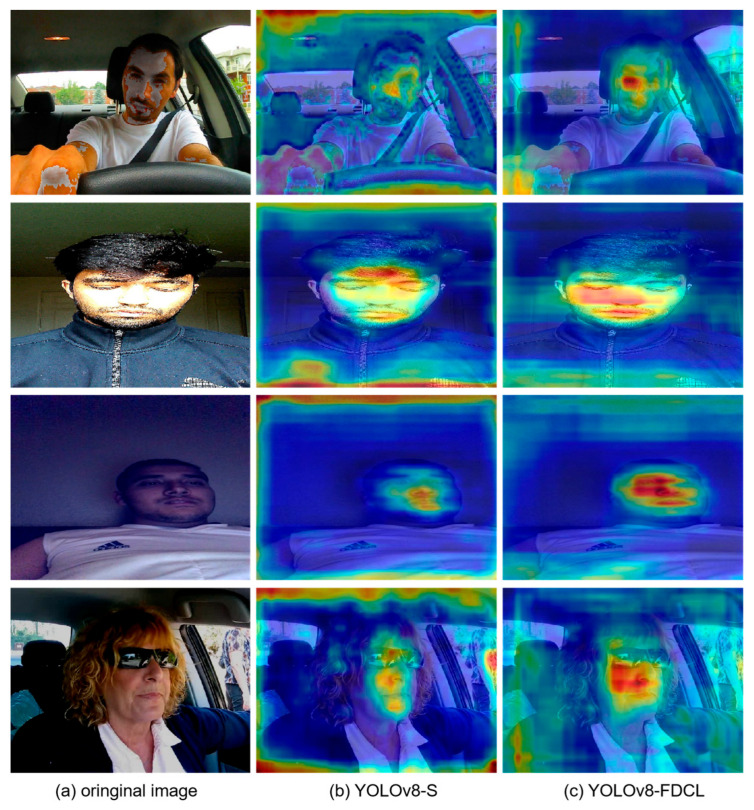
Comparison of heatmap results between YOLOv8-S and YOLO-FDCL algorithms.

To further validate the validity of our model, we performed a visual comparative analysis of the YOLO-FDCL model and the baseline model, as shown in the heat map of Figure 9. After four operations—image sharpening, saturation adjustment, color shift, and brightness adjustment—YOLO-FDCL simulates the complex care environment of the driver in a real driving environment. It can be clearly seen that in the case of the alternation of light caused by the surrounding environment to the driver’s face, our model can focus on the driver’s face better and detect the fatigue state of the baseline, while the baseline model is disturbed by the external environment, and the detection ability is greatly reduced. In Figure 9, our model is still able to accurately identify the driver’s fatigue state when the driving environment is at low brightness. This fully demonstrates the robustness of our model.

## 5. Conclusions

To address the challenges of complex lighting conditions in driver fatigue detection, we propose the YOLO-FDCL algorithm that combines the MobileNetV4 architecture with RepFPN multi-scale feature fusion to enhance fatigue feature capture capability in complex scenes. Experimental results demonstrate that YOLO-FDCL achieves significant improvements of 1.4% in precision (from 97.4% to 98.8%), 1.2% in recall (from 96.3% to 97.5%), 0.8% in mAP@0.5 (from 98.0% to 98.8%), and 1.8% in mAP@0.5:0.95 (from 92.4% to 94.2%) compared to the baseline, proving its practical value in complex driving scenarios. This work addresses the critical lighting robustness challenge that has limited ADAS fatigue detection effectiveness in real-world deployment, enabling automotive manufacturers to deploy reliable fatigue monitoring systems with reduced false alarms and improved detection consistency across diverse driving environments. Although the model demonstrates good accuracy and robustness, limitations remain: reliance on single-frame facial features, lack of temporal modeling for dynamic fatigue evolution, and absence of physiological signal integration. Future research will focus on developing multimodal fusion algorithms that integrate temporal dynamics, physiological features, and vehicle behavior data to enhance generalization performance and practical application value.

## Figures and Tables

**Table 1:** Network architecture of MobileNetV4.

Input	Layer	Block	Output	Stride
640^2^ × 3	Layer0	Conv2D	32	2
320^2^ × 32	Layer1	Fused IB	32	2
160^2^ × 32	Layer2	Fused IB	64	2
80^2^ × 64	Layer3 UIB	ExtraDW	96	2
40^2^ × 96		IB	96	1
40^2^ × 96		IB	96	1
40^2^ × 96		Convnext	96	1
40^2^ × 96	Layer4 UIB	ExtraDW	128	1
20^2^ × 128		ExtraDW	128	1
20^2^ × 128		IB	128	2
20^2^ × 128		IB	128	1
20^2^ × 128		IB	128	1

**Table 2:** Experiment environment configuration.

Laboratory Setting	Configuration Information
CPU	13th Gen Intel Core^TM^ i9-13900H
GPU	NVIDIA GeForce RTX 4070
Operating System	Windows 11
RAM	32 GB
Programming Language	Python 3.8
Deep Learning Framework	Pytorch 2.4.1

**Table 3:** Comparison of advanced detection algorithms on the YAWDD Test.

Method	P	R	mAP_@0.5_	mAP_@0.5:0.95_	Params (M)	FLOPs (G)
Two-stage:						
Faster-RCNN	95.2%	94.1%	95.6%	85.5%	60.745	273.4
Dynamic-RCNN	95.6%	94.5%	96.3%	84.7%	41.35	199.3
One-stage:						
YOLOv5-S	96.9%	96.2%	98.2%	91.5%	7.2	16.5
YOLOv8-S	97.4%	96.3%	98.0%	92.4%	11.14	28.6
YOLOv8-N	97.3%	96.3%	98.1%	91.8%	2.68	6.8
YOLOv8-M	97.2%	96.5%	98.4%	92.8%	23.20	67.4
YOLOv8-L	97.4%	96.8%	98.4%	92.7%	39.43	145.2
YOLOv8-X	97.7%	97.9%	98.5%	92.9%	61.60	226.7
YOLOv10-S	97.1%	96.3%	98.2%	91.4%	7.22	21.4
Deformable-DETR	95.3%	94.2%	96.1%	83.3%	40.1	185.1
RT-DETR	98.3%	95.6%	96.5%	89.8%	31.99	103.4
YOLO-FDCL(ours)	98.8%	97.5%	98.8%	94.2%	10.96	32.3

**Table 4:** Comparison of ablation experiments on different trunks.

	mAP_@0.5_	mAP_@0.5:0.95_	Params (M)	FLOPs (G)
YOLOv8s	98.0%	92.4%	11.14	28.6
YOLOv8s-EfficientVIT	98.3%	92.3%	8.38	20.4
YOLOv8s-Fasternet	98.2%	92.3%	4.17	10.7
YOLOv8s-Starnet	98.4%	92.5%	6.54	17.3
YOLOv8s-Timm	98.2%	92.2%	17.88	46.3
YOLOv8s-MobilenetV4	98.8%	93.3%	10.62	33.9

**Table 5:** Comparison of ablation experiments on different FPN.

	mAP_@0.5_	mAP_@0.5:0.95_	Params (M)	FLOPS (G)
YOLOv8s	98.0%	92.4%	11.14	28.6
YOLOv8s-BiFPN	98.4%	92.4%	7.37	25.0
YOLOv8s-MAFPN	98.3%	92.2%	11.19	31.2
YOLOv8s-HSFPN	98.4%	92.5%	7.13	23.9
YOLOv8s-RepFPN	98.7%	93.4%	9.95	34.5

**Table 6:** Ablation study results.

Model	Mobilenetv4	RepFPN	P	R	mAP_@0.5_	mAP_@50:95_	Params (M)	FLOPs (G)
YOLOv8-S	×	×	97.4%	96.3%	98.0%	92.4%	11.14	28.6
A	√	×	98.3%	96.8%	98.8%	93.3%	10.62	33.9
B	×	√	98.6%	96.4%	98.7%	93.4%	9.95	34.5
YOLO-FDCL	√	√	98.8%	97.5%	98.8%	94.2%	10.96	32.3

**Table 7:** Comparison of advanced detection algorithms on the CLDFD Test.

Model	P	R	mAP_@0.5_	mAP_@50:95_	Params (M)	FLOPs (G)
YOLOv8-S	86.9%	86.4%	0.874	0.81	11,136,374	28.6
YOLO-FDCL	89.7%	88.6%	0.905	0.846	10,955,126	32.3

## Data Availability

The data supporting the findings of this study are available from publicly accessible repositories. The Yawning Detection Dataset (YAWDD) is available at the IEEE Dataport (https://ieee-dataport.org/open-access/yawdd-yawningdetection-dataset, accessed on 11 October 2024). The UTA Real-Life Drowsiness Dataset (UTA-RLDD) is officially hosted by the UTA Vision-Learning-Mining Laboratory and accessible at https://sites.google.com/view/utarldd/home (accessed on 11 October 2024). The Complex Lighting Driving Fatigue Dataset (CLDFD) constructed in this study through systematic augmentation of the aforementioned datasets is available upon reasonable request to the corresponding author. These datasets provide the foundation for the comprehensive analysis and validation of the proposed YOLO-FDCL model under diverse lighting conditions. Further inquiries can be directed to the corresponding author.

## References

[1] Muhammad K., Ullah A., Lloret J., Del Ser J., De Albuquerque V.H.C. (2021). Deep learning for safe autonomous driving: Current challenges and future directions. IEEE Trans. Intell. Transp. Syst..

[2] NHTSA (2022). Overviewof Motor Vehicle Crashes.

[3] Owens J. (2018). Prevalence of Drowsy Driving Crashes: Estimates from a Large-Scale Naturalistic Driving Study.

[4] Fu R., Wang H. (2013). Detection of driving fatigue by using noncontact emg and ecg signals measurement system. Int. J. Neural Syst..

[5] Oviyaa M., Renvitha P., Swathika R., Paul I.J.L., Sasirekha S. Arduino based Real Time Drowsiness and Fatigue Detection for Bikers using Helmet. Proceedings of the 2020 2nd International Conference on Innovative Mechanisms for Industry Applications (ICIMIA).

[6] Fan C., Peng Y., Peng S., Zhang H., Wu Y., Kwong S. (2022). Detection of Train Driver Fatigue and Distraction Based on Forehead EEG: A Time-Series Ensemble Learning Method. IEEE Trans. Intell. Transp. Syst..

[7] Wu E.Q., Xiong P., Tang Z.R., Li G.J., Song A., Zhu L.M. (2022). Detecting Dynamic Behavior of Brain Fatigue Through 3-D-CNN-LSTM. IEEE Trans. Syst. Man Cybern. Syst..

[8] Sandberg D., Wahde M. Particle swarm optimization of feedforward neural networks for the detection of drowsy driving. Proceedings of the 2008 IEEE International Joint Conference on Neural Networks (IEEE World Congress on Computational Intelligence).

[9] Wang M.S., Jeong N.T., Kim K.S., Choi S.B., Yang S.M., You S.H., Lee J.H., Suh M.W. (2016). Drowsy behavior detection based on driving information. Int. J. Automot. Technol..

[10] Zhang F., Dai F. Research and Application of Fatigue Driving Monitoring and Emergency Treatment. Proceedings of the 2017 International Conference on Computer Technology, Electronics and Communication (ICCTEC).

[11] Wang K., Ma Y., Huang J., Zhang C. Driving Performance of Heavy-Duty Truck Drivers under Different Fatigue Levels at Signalized Intersections. Proceedings of the 19th COTA International Conference of Transportation Professionals (CICTP 2019).

[12] Zhang W., Murphey Y.L., Wang T., Xu Q. (2015). Driver Yawning Detection Based on Deep Convolutional Neural Learning and Robust Nose Tracking.

[13] Hsu G., Kang J.H., Huang W.F. (2018). Deep hierarchical network with line segment learning for quantitative analysis of facial palsy. IEEE Access.

[14] Zhu Y., Xie Z., Yu W. (2022). Fatigue Driving Recognition Technology Based on Facial Feature Points in Low-Light Environments. J. Automot. Saf. Energy.

[15] Li X., Li X., Shen Z., Qian G. (2024). Driver fatigue detection based on improved YOLOv7. J. Real Time Image Process..

[16] Liu S., Wang Y., Yu Q., Zhan J., Liu H., Liu J. (2023). A Driver Fatigue Detection Algorithm Based on Dynamic Tracking of Small Facial Targets Using YOLOv7. IEICE Trans. Inf. Syst..

[17] Abe T. (2023). PERCLOS-based technologies for detecting drowsiness: Current evidence and future directions. Sleep Adv..

[18] Chirra V., Reddy U.S., KishoreKolli V. (2019). Deep cnn: A machine learning approach for driver drowsiness detection based on eye state. Rev. d’Intell. Artif..

[19] Alioua N., Amine A., Rziza M. (2014). Driver’s Fatigue Detection Based on Yawning Extraction. Int. J. Veh. Technol..

[20] Zhang F., Wang F. (2020). Exercise fatigue detection algorithm based on video image information extraction. IEEE Access.

[21] Zhuang Q., Kehua Z., Wang J., Chen Q. (2020). Driver Fatigue Detection Method Based on Eye States With Pupil and Iris Segmentation. IEEE Access.

[22] Zhao Z., Zhou N., Zhang L., Yan H., Xu Y., Zhang Z. (2020). Driver Fatigue Detection Based on Convolutional Neural Networks Using EM-CNN. Comput. Intell. Neurosci..

[23] Guo Z., Wang G., Zhou M., Li G. Monitoring and Detection of Driver Fatigue from Monocular Cameras Based on Yolo v5. Proceedings of the 2022 6th CAA International Conference on Vehicular Control and Intelligence (CVCI).

[24] Feng Y., Gong Y., Tu H., Liang J., Wang H. (2020). A Fatigue Driving Detection Algorithm Based on Facial Motion Information Entropy. J. Adv. Transp..

[25] Hu S., Gao Q., Xie K., Wen C., Zhang W., He J. (2024). Efficient detection of driver fatigue state based on all-weather illumination scenarios. Sci. Rep..

[26] Zeng C., Mu Z., Wang Q. (2022). Classifying driving fatigue by using EEG signals. Comput. Intell. Neurosci..

[27] Sheykhivand S., Danishvar S. (2023). Deep Learning for Detecting Multi-Level Driver Fatigue Using Physiological Signals: A Comprehensive Approach. Sensors.

[28] Schmidt E.A., Schrauf M., Simon M., Fritzsche M., Buchner A., Kincses W.E. (2009). Drivers’ misjudgement of vigilance state during prolonged monotonous daytime driving. Accid. Anal. Prev..

[29] Liu C., Wang Y., Zhang K., Chen Y., Zhang C. (2021). A Novel Fatigue Driving State Recognition and Warning Method Based on EEG and EOG Signals. J. Healthc. Eng..

[30] Du G., Li T., Li C., Liu P.X., Li D. (2020). Vision-based fatigue driving recognition method integrating heart rate and facial features. IEEE Trans. Intell. Transp. Syst..

[31] Zhang Z., Ning H., Zhou F. (2022). A systematic survey of driving fatigue monitoring. IEEE Trans. Intell. Transp. Syst..

[32] Sikander G., Anwar S. (2019). Driver Fatigue Detection Systems: A Review. IEEE Trans. Intell. Transp. Syst..

[33] Lin T.Y., Dollár P., Girshick R., He K., Hariharan B., Belongie S. Feature Pyramid Networks for Object Detection. Proceedings of the 2017 IEEE Conference on Computer Vision and Pattern Recognition (CVPR).

[34] Liu S., Qi L., Qin H., Shi J., Jia J. Path Aggregation Network for Instance Segmentation. Proceedings of the 2018 IEEE/CVF Conference on Computer Vision and Pattern Recognition.

[35] Ghiasi G., Lin T.Y., Le Q.V. NAS-FPN: Learning Scalable Feature Pyramid Architecture for Object Detection. Proceedings of the 2019 IEEE/CVF Conference on Computer Vision and Pattern Recognition (CVPR).

[36] Liu S., Huang D., Wang Y. (2019). Learning Spatial Fusion for Single-Shot Object Detection. arXiv.

[37] Tan M., Pang R., Le Q.V. EfficientDet: Scalable and Efficient Object Detection. Proceedings of the 2020 IEEE/CVF Conference on Computer Vision and Pattern Recognition (CVPR).

[38] Guo C., Fan B., Zhang Q., Xiang S., Pan C. AugFPN: Improving Multi-Scale Feature Learning for Object Detection. Proceedings of the 2020 IEEE/CVF Conference on Computer Vision and Pattern Recognition (CVPR).

[39] Qin D., Leichner C., Delakis M., Fornoni M., Luo S., Yang F., Wang W., Banbury C., Ye C., Akin B. (2024). MobileNetV4—Universal Models for the Mobile Ecosystem. European Conference on Computer Vision.

[40] Ding X., Zhang X., Ma N., Han J., Ding G., Sun J. RepVGG: Making VGG-style ConvNets Great Again. Proceedings of the 2021 IEEE/CVF Conference on Computer Vision and Pattern Recognition (CVPR).

[41] Abtahi S., Omidyeganeh M., Shirmohammadi S., Hariri B. YawDD: A yawning detection dataset. Proceedings of the ACM SIGMM Conference on Multimedia Systems.

[42] Ghoddoosian R., Galib M., Athitsos V.A. Realistic Dataset and Baseline Temporal Model for Early Drowsiness Detection. Proceedings of the IEEE/CVF Conference on Computer Vision and Pattern Recognition Workshops.

[43] Zhang H., Chang H., Ma B., Wang N., Chen X. (2020). Dynamic R-CNN: Towards High Quality Object Detection via Dynamic Training. Proceedings of the European Conference on Computer Vision.

[44] Zhao Y., Lv W., Xu S., Wei J., Wang G., Dang Q., Liu Y., Chen J. (2024). DETRs Beat YOLOs on Real-time Object Detection. Proceedings of the 2024 IEEE/CVF Conference on Computer Vision and Pattern Recognition (CVPR).

[45] Zhu X., Su W., Lu L., Li B., Wang X., Dai J. (2020). Deformable DETR: Deformable Transformers for End-to-End Object Detection. arXiv.

